# The current state of forensic imaging– clinical forensic imaging

**DOI:** 10.1007/s00414-025-03464-8

**Published:** 2025-03-18

**Authors:** Fabrice Dedouit, Mathilde Ducloyer, Jamie Elifritz, Natalie L. Adolphi, Grace Wong Yi-Li, Summer Decker, Jonathan Ford, Yanko Kolev, Michael Thali

**Affiliations:** 1 Department of Forensic Pathology, Bâtiment Raymonde Fournet, Place du Dr Baylac, Hôpital Purpan, Toulouse, 31700 France; 2 Department of Forensic Pathology, Nantes University, University Hospital, Bd Jean Monnet, Nantes, F-44000 France; 3 Forensic Radiology Group, Anderson, SC USA; 4 Office of the Medical Investigator, University of New Mexico, Albuquerque, NM 87131 USA; 5 Department of Radiology, Penang General Hospital, Jalan Residensi, Georgetown, 10450 Penang Malaysia; 6 Departments of Radiology and Pathology, University of Southern California Keck School of Medicine, 1450 San Pablo Street, Suite 3500, Los Angeles, CA 90033 USA; 7 Department of General Medicine, Forensic Medicine and Deontology, Medical University - Pleven, 1 St Kliment Ohridski str, Pleven, 5800 Bulgaria; 8 University Zurich, Virtopsy Group, Switzerland

**Keywords:** Forensic imaging, Forensic science, Computed tomography, MRI

## Abstract

Clinical forensic imaging could be defined as the use of imaging first realised for medical care as evidence for a judicial purpose. It requires both forensic experts and clinical radiologists to have a good understanding of imaging modalities and indications and a solid knowledge of the correct terminology. This second part of the review describes the main situations in which imaging may be used for forensic purposes, i.e. blunt trauma, penetrating injuries, asphyxia, physical abuse and neglect.

## Introduction

Every day, emergency departments deal with injured patients who are involved in traumatic situations that require a two-pronged approach: medical and medico-legal. Recommendations and criteria for medicolegal imaging continue to evolve with new epidemiological studies and technological advancements [1, 2]. The selection and combination of imaging modalities requested are best discussed with radiologists while the quality of images and radiation protection depend on the experience and skills of radiology technicians. Yet, formal forensic radiology education and training still lack, particularly in the recognition of forensically relevant radiological signs, interpretation and documentation. Old fractures or minor injuries that can make a difference between “allegation” and “evidence” may be considered insignificant and go unreported by the unsuspecting radiologist, thus negatively impact civil or criminal litigation outcomes. A retrospective study found discrepancies between original radiological reports and expert reports in 18% patients presenting after strangulation and 62% of injured patients [3]. Indeed, clinical radiologists were more focused on reporting medically significant injuries but forensic radiologists tended to include medically insignificant injuries that could be significant in the medicolegal context, such as soft tissue hematomas. Misdiagnosis and missed diagnosis that can lead to radiology malpractice lawsuits are often caused by communication breakdown between radiologists and requesting physicians. The lack clinical information provided upon imaging requests, especially at times of uncertainty [4], also limits radiologists’ roles in early detection of abuse and other intentional injuries.

Implementation and adherence to updated, accredited imaging protocols and guidelines ensure uneventful performance and safe triaging of radiological procedures, production of high-quality images admissible as court evidence as well as objective but accurate pattern recognition of liability-related injuries [5, 6]. Radiology reports should be accurate, concise and easy to understand to the justice system. Effective multidisciplinary team work led by a primary liaison experienced in systematic case coordination and legal matters will increase the reliability and utility different imaging tools as a diagnostic adjuncts and potential court evidence which comply with the Daubert and admissibility criteria of the judicial system [6–10]. And while most of the current literature on this subject is intended as a guide for clinical radiologists and emergency specialists, it provides solid support for medico-legal reasoning and is an essential prerequisite for understanding the mechanism of injuries.

**Fig. 1:**
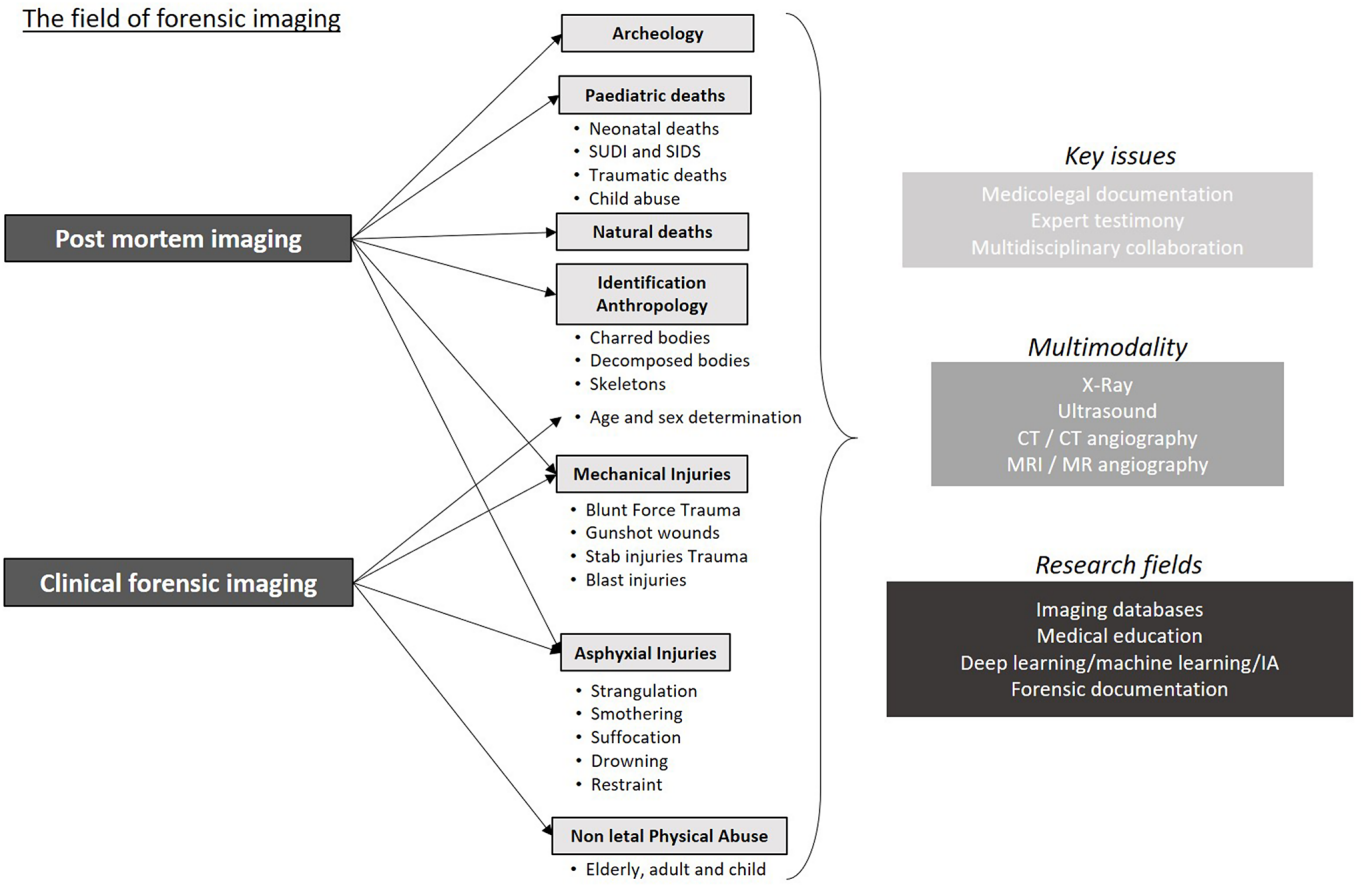
Illustration of the main indications and benefits of post-mortem imaging in the most common forensic situations

The objective of this second part is to present the different applications of different imaging modalities, evidence-based guidelines and recommendations combined from literature review in blunt force trauma, penetrating wounds e.g. gunshot wounds, sharp force trauma, asphyxial abuse and suspected physical abuse in adults. Figure 1 summarises these different indications, some of which are common to post-mortem imaging. In the final paragraph, it discusses the specific role of forensic imaging in court.

## Recommended radiological tools and uses in clinical forensic context

The main role of imaging is to document injury types, severity as well as patterns that are medicolegally relevant, which can confirm or refute the postulated mechanism and timing of injury [4, 10, 11]. The differentiation between inflicted and accidental trauma can be difficult, when there are confounding natural diseases and comorbidities (such as the scenario where risk of fracture could be related to malnutrition and/or osteoporosis) [4, 10, 11]. Good clinical history, relevant examination findings, trauma mechanism and suspicious injury circumstances, if communicated to radiologist, are likely to increase sensitivity and specificity for suspicious trauma, abuse and mechanism mismatch detection. The advantages or disadvantages of each imaging modality enables appropriate modality selection for each case.

### Blunt force trauma

Blunt force trauma can be a result of accidents e.g. road traffic accidents, interpersonal violence e.g. assault or self-inflicted e.g. fall from height. Victims or/and perpetrators either present with injuries to primary care or emergency departments. Primary survey and stabilization should precede any advanced imaging and imaging does not supersede careful evidence collection and medical work up by trained healthcare personnel [12–18].

Three main imaging modalities are used in blunt force injuries: US, X-rays and CT. Rapid bedside focused assessment with sonography in trauma (FAST), extended FAST (eFAST) and/or chest ultrasounds are quick, preliminary radiation-free tools used to detect hemoperitoneum, hemopericardium, haemothorax, pneumothorax, pericardial fluid or tamponade in primary and secondary surveys [13–15]. False negatives have been reported [12, 17]. Hence it is pertinent for the physician to be alert for signs of hemodynamically instability. The development of contrast enhanced ultrasound allows all vascular phases to be assessed in real time, increasing detection of parenchymal and vascular injuries [18]. Sensitivity and specificity of contrast enhanced ultrasound have been reported to be comparable with CECT with the advantage of reduced radiation exposure [19].

The utilization of ultrasonography for diagnosing nasal bone fractures demonstrates high specificity and sensitivity, which, along with its accessibility, makes it significant in a medicolegal context [20, 21]. Radiographic examination is minimally informative for detecting fractures of the lateral nasal bones, whereas CT scans are rarely used for isolated nasal injuries. The use of US is also promising for diagnosing fractures of other facial bones, as well as subcutaneous hematomas and various musculoskeletal injuries [22].

Portable supine anteroposterior chest (CXR) and pelvic radiographs should be reserved for polytrauma patients too unstable or critically ill for CT due to lower sensitivity for internal injuries Chest radiographs may be performed as part of the primary survey in adults with severe respiratory compromise, suspected lung haemorrhage, haemothorax, tension pneumothorax, pneumomediastinum or pneumopericardium [17]. Anteroposterior radiographs are less sensitive than posteroanterior radiographs for rib fractures and up to 50% of rib fractures can be missed at radiography, even when oblique rib views are obtained [23–25]. Dedicated rib views are not recommended unless needed to provide legal documentation of injury [26]. In stable patients with low energy or isolated trauma, radiographs at the region of trauma, with or without ultrasound are the first line of imaging [17]. ]. In children less than 16-years-old, 20% of complex injuries may appear normal on pelvic radiographs and CT due to the relative elasticity of bones and reassembly of the pelvic skeleton even with roll-over traumas [12]. The absence of skeletal fractures in children should not be presumed as low energy impact, but the presence of skeletal fractures indicates high energy trauma [27].

CT remains the most practical, specific and sensitive diagnostic tool (the gold standard) for identification of traumatic bone, solid organ, hollow viscus, lung and vascular injuries in the acute setting [14, 28]. Contrast enhanced CT (CE-CT) is recommended in stable patients [14, 17, 28]. CT in children should be limited to areas where assessment is required, considering the radiation risks. In patients with major trauma, FAST, CXR and other imaging are not necessary if immediate CT is available [17]. CT angiogram (CTA) is an additional non-invasive technique used to localize sources of active haemorrhage, traumatic vascular complications e.g. pseudoaneurysm or arteriovenous fistulas or to follow up diagnosed bleeding cases that are treated conservatively [29]. Cases should be evaluated first by the vascular team prior to advanced imaging. Positive scans may indicate the need for minimally invasive catheter angiogram and embolization by interventional radiology team in cases of (i) transient hemodynamic response with abdominal solid organ injury, (ii) stable great vessel injury, (iii) penetrating proximal vessel injury, (iv) unstable pelvic injury with haemorrhage difficult to compress or manage with surgical methods and (v) no further life-threatening injury [30].

### Penetrating injuries

Portable radiographs provide a preliminary overview of projectile, foreign body or fragment locations and detect free intracranial, intrathoracic, intraabdominal and subcutaneous air in unstable patients.

In stab wounds, penetrating weapons retained in the body may help tamponade active haemorrhages temporarily and should not be removed prior to imaging. Schmidt reported that 45.9% of survivors of sharp force trauma had defensive injuries [31]. In this study cohort, injury locations were reported to be 63.7% on the left side, 15.3% head, 15.3% neck, 45.9% thorax, 11.1% abdomen, 6.3% lumbar and gluteal, 6.1% lower extremity injuries respectively.

Several studies have described high sensitivity and specificity of thin-cut CT with 3D-multiplanar reformats (3D-MPR) for visualization of foreign bodies and injuries [32, 33]. CECT Whole Body and CT angiography (CTA) may be indicated when sources of bleeding or injury are unknown [18]. CTA is highly sensitive and specific for example in traumatic neck vascular injuries such as dissecting flaps, mural thrombosis, vessel occlusion and pseudoaneurysm. CTA also aids planning for conventional catheter angiogram and subsequent endovascular repair or stenting.

In gunshot injuries, CT helps estimate the number and location of projectiles and bone fragments, identifying bullet embolization, direct and indirect soft tissue injuries, as well as lung and bone injuries due to shock waves and cavitation. Adhesive metallic skin markers help mark the entry and exit wound sites, and are invaluable for trajectory analysis [33]. Bullet paths are curvilinear rather than linear in most cases and may diverge with distance, depending on the tissues they interact with and the rotation of the bullet nose from the line of flight (bullet yaw). Knowledge on ballistics and mechanism of firearm injuries increases the accuracy of trajectory analysis on imaging. Several reviews have proposed in depth management algorithms for gunshot wounds [33, 34]. Similar to blunt force trauma, MRI may be indicated in cases of spinal cord and ligamentous injuries with progressive neurological deterioration. There is better prognosis for gunshot wounds below the conus medullaris compared to above due to more spinal canal space at this level [35]. Safety depends on the clinical presentation and projectile properties. Non-jacketed, non-ferromagnetic bullets, fragments containing copper or aluminium jackets or projectiles made of titanium, stainless steel and aluminium as well as small copper fragments may not cause significant complications e.g. migration and heating [35]. However, in most acute settings, the ferromagnetic properties of projectiles are often unknown. Preceding radiographs and CT are unable to differentiate ferromagnetic and non-ferromagnetic projectiles. Nevertheless, complete survey, CT and multidisciplinary review should be done prior to MRI [34]. The risks of bullet migration, black void artifacts and heating depend on the degree of ferromagnetism, mass, location, shape, orientation, shape of the projectile that surround it. The risks of bullet migration, black void artifacts and heating depend on the degree of ferromagnetism, mass, location, shape, and orientation of the projectile. Tissue scarring may provide resistance against the magnetic field, preventing displacement and fragmentation of bullets embedded in tissue or bone [36].

### Asphyxia injuries with focus on strangulation and ligature hanging

Asphyxial injuries may be either inflicted or self-inflicted. Inflicted harm includes intimate partner or sexual violence, domestic violence including elderly abuse, policing tactics or refugee torture. Self-inflicted-injuries may be caused by intent for suicide, insurance fraud or sexual stimulation. Symptoms vary in intensity and timing (immediate or delayed), depending on the duration, degree and location of pressure, ligature material, shearing forces and forced neck extension. Minimal pressure on the neck can cause internal injury without detectable external injuries [37]. The main mechanisms of injuries are airway and vascular obstruction to vital structures, cartilage and soft tissue injuries as well as subsequent neurological, cardiac and pulmonary complications such as delayed pulmonary oedema.

Plain radiographs lack sensitivity and specificity in evaluating vascular and soft tissue structures and hence are not recommended [8, 9]. Carotid Doppler ultrasound is also not recommended in screening for dissections unless CT is not available, due to sonographic inaccessibility of the upper carotid and vertebral arterial segments and operator dependence [8, 9, 38, 39]. Combination of non-contrast CT brain and CTA of the head and neck are the modalities of choice for assessment of vascular occlusions, dissections and pseudoaneurysm but may be omitted in patients with no significant neurologic deficit and low suspicion for cerebrovascular injury [2, 8, 29, 40, 41]. MRI or CECT Neck can be performed in suspected cartilage, laryngeal trauma and cervical injuries, neck hematomas as well as chronic complications such as neck abscesses [3, 42–45].

A retrospective analysis by Ruder et al. found a substantial number of unreported soft tissue hematomas and larynx fractures were missed on CT compared to MRI [3]. Overall sensitivity and specificity of original radiology reports were 78% and 97% on MRI and 30% and 98% for CT. The clinical recommendation was that MRI was preferred over CT for the investigation of alert patients because of higher accuracy in addition to protection of the typically younger patient population to ionizing radiation. Heimer et al. also concurred that MRI neck could reveal internal injuries that could not be detected externally, but had no additional value in estimation of severity of non-fatal strangulation [41].

### Physical abuse & neglect

The main clinical challenge is to differentiate physical abuse or neglect from accidental trauma or natural disease that predispose to traumatic injuries. Types of injuries may include blunt force trauma, penetrating and asphyxial injuries. The role of imaging is either to (i) Supplement history, clinical examinations and laboratory investigations in criminal investigations or (ii) Increase detection of incidental fracture patterns that may raise suspicion of child or adult abuse; both of which can have similar and overlapping imaging findings.

Effective communication between the attending primary physician and radiologists is pertinent to increase the detection rates of physical abuse [10]. Victims of abuse tend to present more than a day after injury. Children less than 2 years old are unable to vocalize areas of injury and hence whole-body skeletal surveys (radiographs) are performed to detect recent and older fractures. In older children and adults, imaging is more selective, typically targeted at suspected bone fracture locations [46]. Incomplete history and examination could lead to misses in detecting old, healed but suggestive fractures. Regarding datation of the trauma, and specifically bone trauma, works dealing with quantitative MRI showed promising results [47].

Useful histories include complains of assault, injury mechanism, object impressions, superficial injuries, genital and perianal findings, signs of neglect, risk factors and demographics of both victim and perpetrators e.g. drug or alcohol misuse, incompatible histories, clinical findings and results from screening assessment tools [48–52]. Additional screening tests, including forearm, maxillofacial and chest imaging, have been suggested to be analogous to skeletal survey for these patients, particularly the elderly who are cognitively impaired [53, 54].

A retrospective study of 92 patients found more common locations for elderly physical abuse were the head and neck (30.4%), upper extremity (37%) and chest (30.4%) [46]. A study in elder abuse noted bruises tend to be larger (> 5 cm) versus accidental bruises [55]. More specifically, suspicious fractures, combined with suggestive history and external findings e.g. defense wounds, were mostly multiple; involving posterior ribs, distal and diaphyseal long bone [46, 54, 56]. There was also high incidence of old fractures (19.6%), joint dislocations (12%) and soft tissue lesions (54.3%) [46]. Intentional injuries tend to be more proximal or central, whilst accidental trauma tends to be more distal. In a prospective study of 78 cases of elderly abuse, physical abuse victims were less likely to have fractures (8% vs. 22%) or lower extremity injuries (9% vs. 41%); and significantly more likely to have bruising (78% vs. 54%), and maxillofacial, dental and neck injuries (67% vs. 28%) compared to unintentional fallers [7]. CT or MRI of the brain may be considered even in minor head trauma in the elderly, which leads to morbidity, mortality and functional decline.

Midface and zygomatic fractures, more accessible to assault, as well as distal upper extremity injuries i.e. defensive injuries can also be seen in intimate partner violence and domestic abuse. The left side of face and middle 1/3 of the face, particularly nasal injuries, are more common as 90% of the population are right-handed [57, 58]. Pregnant victims of intimate partner violence can present with complications to both mother and foetus [59, 60]. These patients also tend to return for imaging more frequently at a median of 4 exams per patient versus 1 exam per patient in the control group Non-contrast MRI can be done in pregnancy if indicated. For more severe injuries requiring CT imaging for pre-operative planning, radiation dose should ideally be limited to < 50 mGyr with an abdominal shield where possible [61].

### Place of imaging in courts

Giving expert testimony requires a number of rules. The most important is to be able to justify one’s conclusions and to know the limits of the tools or methods used. For example, considering that a morphological variation or a particular feature is rare enough to be the basis of an identification is a subjective judgement. Experts should keep in mind that some morphological features of bone, such as fractures, pathological conditions and surgical material, which could be thought to be rare, may in fact be quite common [62]. Thus, the radiologist, forensic pathologist or anthropologist must be aware of the Daubert standard when performing this type of expertise [63].

The guidelines of the Daubert standard highlight numerous questions pertaining to the nature of science and the role of the expert witness in providing testimony [63]. These include inquiries into the fundamental theory and its empirical validation, the existence of standards regulating the technique, the extent to which the theory or technique has undergone peer review and publication, the known or potential error rate, the general acceptance of the theory, the extent to which the expert has considered alternative explanations, and the justification for extrapolating from an accepted premise to an unsubstantiated conclusion. These are indeed numerous questions that should be answered by the expert conducting an anthropological assessment or comparative identification. It is evident that the methods employed for reconstructive identification are derived from a population sample that is typically distinct from the geographical origin or socio-economic status of the individual under study. The underlying issue is consistently the same: adapting disparate population data to a singular individual. This is where statistics, and Bayesian statistics in particular, become invaluable. It enables the answering of various questions, particularly those relating to age assessment, with probability. This type of answer appears to be the most “scientifically honest” and respectful of the Daubert rules. Prior to 1993, federal and most state courts followed the 1923 Frye standard of “general acceptance” in the relevant scientific field.

A number of district court rulings and appellate decisions have been based on the grounds that the proposed testimony did not meet the Daubert standard of scientific knowledge. This was due to the fact that the methodology was untested, there was a lack of peer review and publication, a potentially high error rate, and that the conclusions were not generally accepted in the scientific community. As a result, the experts’ conclusions were rejected.

Apart from the absolute priority for a forensic expert to be able to justify his or her conclusions, forensic imaging can also enter the courtroom in a variety of ways.

There is general agreement that visual representations of 3D data can add value to the presentation and understanding of complex evidence, even if 3D models and simulations can have biasing effects if not used properly [64]. Previous studies have used mock juries to investigate the effects of using digital and physical 3D models on individuals’ learning and decision-making processes [65, 66]. Blau et al. supplemented fictional pathology expert testimony with a range of visual aids, including photographs, CT images and a 3D print, and found that testimony using the 3D print was rated as the easiest to understand by study participants [67]. Dunn et al. tested the influence of animation on the interpretation of events by study participants and found that, even when presenting identical facts, animation affected people’s perceptions of them [68].

The possibility of showing forensic 3D printing in court is interesting. Baier et al. have shown that the choice of printing technology is a variable with a potentially negative impact on overall accuracy. It has already been established that different 3D printing hardware can produce different results [69]. Therefore, in order to use a 3D printed object as evidence in court, it is necessary to verify the exact technology used to produce it. For bone trauma, Baier recommended using an established printer (Material Jetting with fully enveloping, water-soluble support structures, which produced the best results in her study) and verifying the print against the original, ideally by surface scanning and surface-to-surface comparison.

3D digital evidence is a powerful tool to add clarity to expert testimony and reduce potential confusion surrounding scientific or medical evidence, thus providing the jury with the best possible tools to reach an informed verdict. The use of a 3D printed object as a demonstrative aid in a murder trial enabled the lawyers in the case presented to hold the jury’s attention, and potentially improve their understanding of the pathological facts. It also allowed the court to demonstrate that it had arrived in the 21st century, which maintains its credibility in the public eye. However, physical models should be used with caution and only where they add to existing evidence to avoid the unjustified risk of cognitive bias [69].

## Conclusion

Clinical radiology takes an important place in the management of injured patients, as it can constitute crucial evidence for the judicial procedure, by depicting the exact nature of the injuries and helping to understand their mechanism. The patient may recover and its wounds disappear, imaging will provide a reliable and permanent record of the victim’s state of health at the time of the incident. Forensic pathologists should be aware of the potential for clinical imaging to serve as an objective tool for describing injuries sustained by a living victim. Clinical radiologists, for their part, should always consider the possibility of their conclusions and reports being used as evidence in a judicial inquiry.

## Data Availability

Non-applicable (review).

## Code availability

Non-applicable (review).
